# Role of SGK1 in the Osteogenic Transdifferentiation and Calcification of Vascular Smooth Muscle Cells Promoted by Hyperglycemic Conditions

**DOI:** 10.3390/ijms21197207

**Published:** 2020-09-29

**Authors:** Florian Poetsch, Laura A. Henze, Misael Estepa, Barbara Moser, Burkert Pieske, Florian Lang, Kai-Uwe Eckardt, Ioana Alesutan, Jakob Voelkl

**Affiliations:** 1 Institute for Physiology and Pathophysiology, Johannes Kepler University Linz, Altenberger Strasse 69, 4040 Linz, Austria; florian.poetsch@jku.at (F.P.); barbara.moser@jku.at (B.M.); jakob.voelkl@jku.at (J.V.); 2 Department of Internal Medicine and Cardiology, Charité—Universitätsmedizin Berlin, Campus Virchow-Klinikum, Augustenburger Platz 1, 13353 Berlin, Germany; laura.henze@charite.de (L.A.H.); misael.estepa@gmail.com (M.E.); burkert.pieske@charite.de (B.P.); 3 Berlin Institute of Health (BIH), Anna-Louisa-Karsch 2, 10178 Berlin, Germany; 4 German Centre for Cardiovascular Research (DZHK), Partner Site Berlin, 13347 Berlin, Germany; 5 Department of Internal Medicine and Cardiology, German Heart Center Berlin (DHZB), Augustenburger Platz 1, 13353 Berlin, Germany; 6 Department of Physiology I, Eberhard-Karls University, Wilhelmstr. 56, 72076 Tübingen, Germany; florian.lang@uni-tuebingen.de; 7 Department of Nephrology and Medical Intensive Care, Charité—Universitätsmedizin Berlin, Campus Virchow-Klinikum, Augustenburger Platz 1, 13353 Berlin, Germany; kai-uwe.eckardt@charite.de

**Keywords:** vascular calcification, vascular smooth muscle cells, osteogenic transdifferentiation, diabetes mellitus, high glucose, advanced glycation end products, SGK1, NF-κB

## Abstract

In diabetes mellitus, hyperglycemia promotes the osteogenic transdifferentiation of vascular smooth muscle cells (VSMCs) to enhance medial vascular calcification, a common complication strongly associated with cardiovascular disease and mortality. The mechanisms involved are, however, still poorly understood. Therefore, the present study explored the potential role of serum- and glucocorticoid-inducible kinase 1 (SGK1) during vascular calcification promoted by hyperglycemic conditions. Exposure to high-glucose conditions up-regulated the SGK1 expression in primary human aortic VSMCs. High glucose increased osteogenic marker expression and activity and, thus, promoted the osteogenic transdifferentiation of VSMCs, effects significantly suppressed by additional treatment with the SGK1 inhibitor EMD638683. Moreover, high glucose augmented the mineralization of VSMCs in the presence of calcification medium, effects again significantly reduced by SGK1 inhibition. Similarly, SGK1 knockdown blunted the high glucose-induced osteogenic transdifferentiation of VSMCs. The osteoinductive signaling promoted by high glucose required SGK1-dependent NF-κB activation. In addition, advanced glycation end products (AGEs) increased the SGK1 expression in VSMCs, and SGK1 inhibition was able to interfere with AGEs-induced osteogenic signaling. In conclusion, SGK1 is up-regulated and mediates, at least partly, the osteogenic transdifferentiation and calcification of VSMCs during hyperglycemic conditions. Thus, SGK1 inhibition may reduce the development of vascular calcification promoted by hyperglycemia in diabetes.

## 1. Introduction

The high mortality of patients with diabetes mellitus is strongly associated with cardiovascular complications [1,2]. Diabetes is considered a major independent risk factor for cardiovascular diseases [1], which are a leading cause of death in diabetic patients [1]. A key role in the development of cardiovascular diseases in diabetes is attributed to medial vascular calcification [2]. Vascular calcification may increase vascular stiffness and pulse pressure, leading to impaired organ perfusion, cardiac hypertrophy, as well as diastolic dysfunction [3,4]. Accordingly, vascular calcification is strongly associated with the mortality of patients [4,5]. Nonetheless, no efficient therapeutic strategies to prevent or reduce vascular calcification are available so far [4,6,7].

Vascular calcification is accelerated in patients with diabetes [2,8,9,10], but the underlying mechanisms are still incompletely understood. Diabetic nephropathy is a prevalent severe complication of these patients [9] and kidney disease triggers excessive vascular calcification through a combination of many pathological factors, especially the dysregulation of mineral homeostasis [4,7,11,12,13]. In diabetic patients, vascular calcification is increased even before the development of nephropathy, however to a lower extent than in diabetic patients with renal disease [14]. Hyperglycemia was described to play a crucial role in the pathophysiology of vascular calcification [15]. High glucose was suggested to directly promote vascular calcification [15,16,17]. In addition, hyperglycemia may lead to the glycation of proteins and lipids and the increased formation of advanced glycation end products (AGEs) [18], additional key factors that drive vascular calcification [18,19,20,21].

Vascular calcification involves an active cell-mediated process [7]. During vascular calcification, the expression of osteogenic transcription factors such as core-binding factor α 1 (CBFA1) is increased in vascular smooth muscle cells (VSMCs), which up-regulate the expression of osteoblast specific proteins and, therefore, lead to a phenotypical switch to VSMCs with osteoblast-like characteristics [7,22]. The osteogenic transdifferentiation of VSMCs is essential for the initiation and progression of vascular calcification [7,23]. CBFA1 deficiency in VSMCs was shown to protect from vascular calcification [24]. Moreover, a key osteogenic enzyme in this process is tissue-nonspecific alkaline phosphatase (ALPL), which cleaves the endogenous calcification inhibitor pyrophosphate to allow unrestrained calcification [7,25]. Hyperglycemia and AGEs promote the osteogenic transdifferentiation of VSMCs to enhance vascular calcification [15,16,19,20], but the cellular mechanisms involved are still elusive.

The transcription factor NF-κB (nuclear factor kappa-light-chain-enhancer of activated B-cells) emerged as a critical regulator of vascular calcification [26]. NF-κB is activated by the inhibitor of nuclear factor kappa B (IkB) kinase (IKK) complex, consisting of two kinases IKKα and IKKβ and a regulatory subunit IKKγ [27]. The serum- and glucocorticoid-inducible kinase 1 (SGK1) phosphorylates IKKα to activate NF-κB, an effect requiring IKKβ [28]. Interestingly, IKKβ also serves a kinase-independent function, suppressing vascular calcification through β-catenin inactivation [29]. The SGK1 is a key component of intracellular signaling, regulating the osteo-/chondrogenic transdifferentiation and calcification of VSMCs during hyperphosphatemic [7,30,31] and pro-inflammatory [7,32] conditions. SGK1 promotes osteo-/chondrogenic transdifferentiation via NF-κB activation [30], which in turn up-regulates the expression of CBFA1 and subsequently of ALPL [30,33], as well as the expression of RNA-destabilizing protein tristetraprolin (encoded by the *ZFP36* gene), which reduces the mRNA levels of *ANKH*, a protein involved in extracellular pyrophosphate homeostasis [30,34]. More importantly, the inhibition or deficiency of SGK1 is able to reduce vascular calcification in vitro and in vivo [30,32].

SGK1 expression is up-regulated in VSMCs following exposure to high glucose levels [30]. Moreover, both high glucose [35,36,37] and AGEs [18] were shown to activate NF-κB in the vasculature, which further plays a critical role in diabetic vascular dysfunction [35]. Therefore, the present study investigated the potential role of SGK1-dependent signaling in the osteogenic transdifferentiation and calcification of VSMCs triggered by hyperglycemic conditions in vitro.

## 2. Results

**Figure 1:**
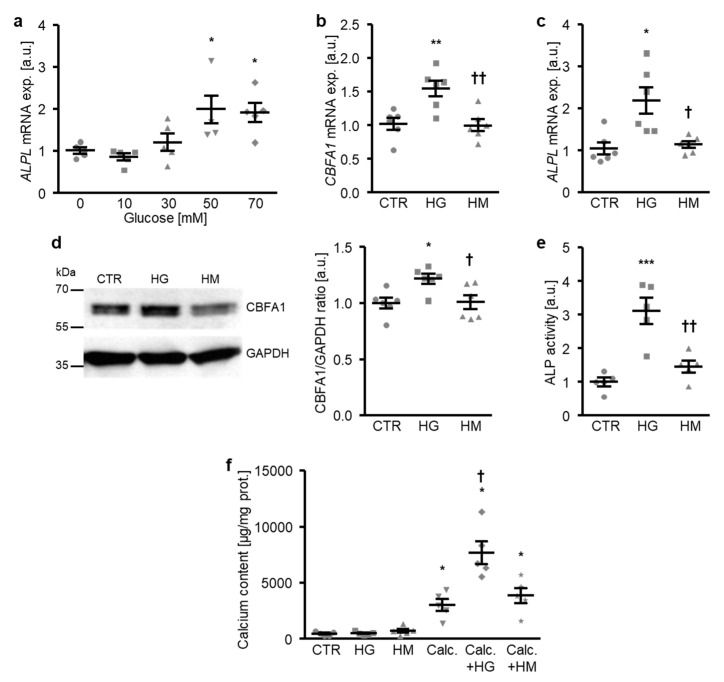
High glucose promotes the osteogenic transdifferentiation and calcification of HAoSMCs. (**a**) Scatter dot plots and arithmetic means ± SEM (*n* = 5; arbitrary units, a.u.) of the *ALPL* relative mRNA expression in HAoSMCs following treatment with the indicated concentrations of glucose (0–70 mM). (**b**,**c**) Scatter dot plots and arithmetic means ± SEM (*n* = 6; a.u.) of the *CBFA1* (**b**) and *ALPL* (**c**) relative mRNA expression in HAoSMCs following treatment with control (CTR), 50 mM of glucose (HG), or 50 mM of mannitol (HM). (**d**) Representative original Western blots and scatter dot plots and arithmetic means ± SEM (*n* = 6; a.u.) of the normalized CBFA1/GAPDH protein ratio in HAoSMCs following treatment with control (CTR), 50 mM of glucose (HG), or 50 mM of mannitol (HM). (**e**) Scatter dot plots and arithmetic means ± SEM (*n* = 5, a.u.) of the ALP activity in HAoSMCs following treatment with control (CTR), 50 mM of glucose (HG), or 50 mM of mannitol (HM). * *p* < 0.05, ** *p* < 0.01, *** *p* < 0.001 significant vs. control HAoSMCs; † *p* < 0.05, †† *p* < 0.01 significant vs. HG-treated HAoSMCs. (**f**) Scatter dot plots and arithmetic means ± SEM (*n* = 5, µg/mg protein) of the calcium content in HAoSMCs following treatment with control (CTR) or calcification medium (Calc.) without and with 50 mM of glucose (HG) or 50 mM of mannitol (HM). * *p* < 0.05 significant vs. control HAoSMCs; † *p* < 0.05 significant vs. Calc.-treated HAoSMCs.

To investigate the mechanisms involved in the osteogenic transdifferentiation and calcification of VSMCs during hyperglycemic conditions, a first series of experiments was performed in primary human aortic smooth muscle cells (HAoSMCs) treated with increasing concentrations of glucose. As a result, the addition of glucose to the cell culture medium up-regulated the *ALPL* mRNA expression in HAoSMCs in a dose-dependent manner, an effect reaching statistical significance at a 50 mM glucose concentration (Figure 1a). Treatment with high glucose, but not equal concentrations of mannitol as osmotic control, significantly increased osteogenic transcription factor *CBFA1* mRNA and protein expression (Figure 1b,d), as well as the *ALPL* mRNA expression and ALP activity (Figure 1c,e) in HAoSMCs, thus, promoting osteogenic transdifferentiation. Furthermore, high glucose did not strongly modify the calcification of HAoSMCs during control conditions, but significantly augmented the calcium deposition of HAoSMCs in the presence of calcification medium containing high phosphate and calcium levels as substrates for mineralization (Figure 1f). In contrast, high mannitol treatment did not affect calcification of HAoSMCs during either control or pro-calcific conditions (Figure 1f). Thus, exposure to high glucose concentrations induced the osteogenic transdifferentiation and calcification of HAoSMCs—effects mediated by mechanisms, mainly, other than osmolality changes.

**Figure 2:**
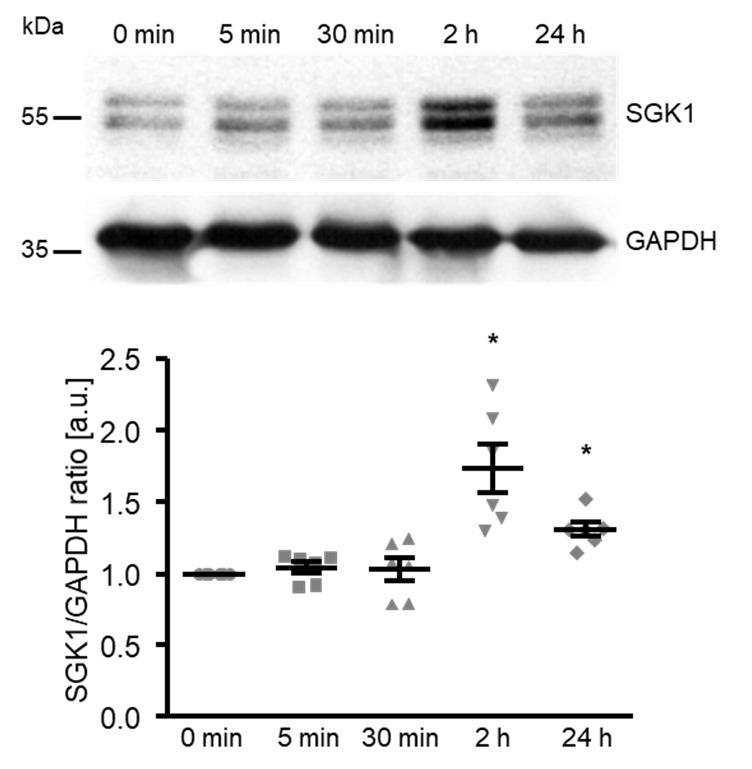
High glucose up-regulates the SGK1 protein abundance in HAoSMCs. Representative original Western blots and scatter dot plots and arithmetic means ± SEM (*n* = 6; arbitrary units, a.u.) of the normalized SGK1/GAPDH protein ratio in HAoSMCs following treatment for the indicated time (0–24 h) with 50 mM of glucose. * *p* < 0.05 significant vs. control HAoSMCs.

To elucidate the underlying mechanisms of the high glucose-induced osteogenic transdifferentiation and calcification of HAoSMCs, the next experiments explored the effects on SGK1 expression. As shown by Western blotting, high glucose significantly up-regulated the SGK1 protein abundance following 2 h of treatment, the levels remaining significantly higher after up to 24 h of treatment (Figure 2).

**Figure 3:**
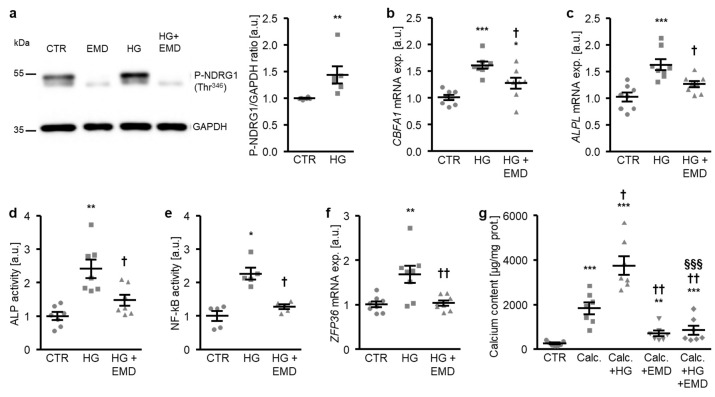
Inhibition of SGK1 blunts high glucose-induced osteogenic signaling and the calcification of HAoSMCs. (**a**) Representative original Western blots (*n* = 6) showing phospho-NDRG1 (Thr^346^) and GAPDH protein abundance in HAoSMCs following treatment with control (CTR) or 50 mM of glucose (HG) without and with 50 µM of SGK1 inhibitor EMD638683 (EMD). Scatter dot plots and arithmetic means ± SEM (*n* = 6; arbitrary units, a.u.) of the normalized phospho-NDRG1 (Thr^346^)/GAPDH protein ratio in HAoSMCs following treatment with control (CTR) or 50 mM of glucose (HG). (**b**,**c**) Scatter dot plots and arithmetic means ± SEM (*n* = 8; a.u.) of the *CBFA1* (**b**) and *ALPL* (**c**) relative mRNA expression in HAoSMCs following treatment with control (CTR) or 50 mM of glucose (HG) without or with 50 µM of SGK1 inhibitor EMD638683 (EMD). (**d**) Scatter dot plots and arithmetic means ± SEM (*n* = 7, a.u.) of the ALP activity in HAoSMCs following treatment with control (CTR) or 50 mM of glucose (HG) without or with 50 µM of SGK1 inhibitor EMD638683 (EMD). (**e**) Scatter dot plots and arithmetic means ± SEM (*n* = 5; a.u.) of the NF-κB-dependent transcriptional activity in HAoSMCs following treatment with control (CTR) or 50 mM of glucose (HG) without or with 50 µM of SGK1 inhibitor EMD638683 (EMD). (**f**) Scatter dot plots and arithmetic means ± SEM (*n* = 8; a.u.) of the *ZFP36* relative mRNA expression in HAoSMCs following treatment with control (CTR) or 50 mM of glucose (HG) without or with 50 µM of SGK1 inhibitor EMD638683 (EMD). * *p* < 0.05, ** *p* < 0.01, *** *p* < 0.001 significant vs. control HAoSMCs; † *p* < 0.05) †† *p* < 0.01 significant vs. HG-treated HAoSMCs. (**g**) Scatter dot plots and arithmetic means ± SEM (*n* = 7, µg/mg protein) of the calcium content in HAoSMCs following treatment with control (CTR) or calcification medium (Calc.) alone and together with 50 mM of glucose (HG) and without and with 50 µM of SGK1 inhibitor EMD638683 (EMD). ** *p* < 0.01, *** *p* < 0.001 significant vs. control HAoSMCs; † *p* < 0.05, †† *p* < 0.01 significant vs. Calc.-treated HAoSMCs; §§§ *p* < 0.001 significant between Calc. + HG and Calc. + HG + EMD-treated HAoSMCs.

A further series of experiments investigated whether SGK1 plays a role in osteogenic signaling promoted by high glucose in HAoSMCs. To this end, HAoSMCs were treated with control and high glucose in the presence or absence of the SGK1 inhibitor EMD638683. As shown in Figure 3a, the high glucose treatment significantly increased the phosphorylation of NDRG1 at Thr^346^, a direct target of SGK1 as a marker for SGK1 activity [30,38]. Additional treatment with the SGK1 inhibitor suppressed NDRG1 phosphorylation at Thr^346^ during both control and high glucose conditions (Figure 3a). The high glucose-induced *CBFA1* and *ALPL* mRNA expression as well as ALP activity were all significantly blunted in the presence of the SGK1 inhibitor (Figure 3b–d). In addition, high glucose triggered SGK1-dependent osteoinductive signaling in HAoSMCs. High glucose induced the activation of the transcription factor NF-κB and the downstream target *ZFP36* mRNA expression, effects significantly suppressed by SGK1 inhibition (Figure 3e,f). Furthermore, additional treatment with the SGK1 inhibitor significantly reduced the mineralization of HAoSMCs triggered by high glucose during pro-calcific conditions (Figure 3g). In accordance with previous findings, SGK1 inhibition blunted the calcification of HAoSMCs promoted by calcification medium alone (Figure 3g). Taken together, SGK1-dependent osteoinductive signaling mediated, at least in part, the osteogenic transdifferentiation and calcification of HAoSMCs triggered by high glucose concentrations.

**Figure 4:**
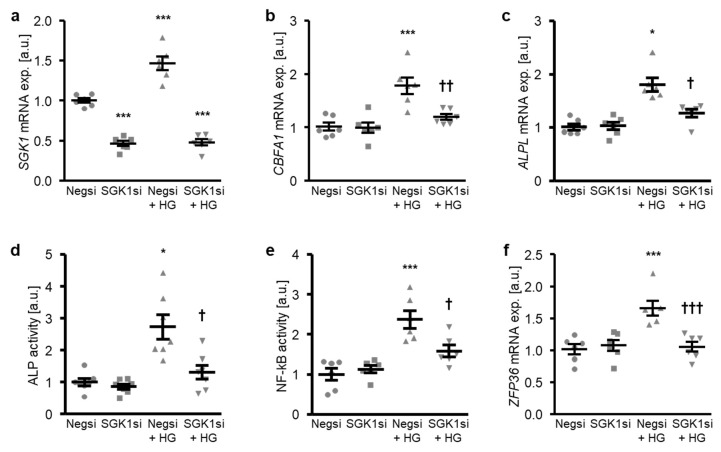
Silencing of SGK1 inhibits high glucose-induced osteogenic signaling in HAoSMCs. (**a**–**c**) Scatter dot plots and arithmetic means ± SEM (*n* = 6; arbitrary units, a.u.) of the *SGK1* (**a**), *CBFA1* (**b**), and *ALPL* (**c**) relative mRNA expression in HAoSMCs following transfection with negative control siRNA (Negsi) or SGK1 siRNA (SGK1si) and treatment with control or 50 mM of glucose (HG). (**d**) Scatter dot plots and arithmetic means ± SEM (*n* = 7, a.u.) of the ALP activity in HAoSMCs following transfection with negative control siRNA (Negsi) or SGK1 siRNA (SGK1si) and treatment with control or 50 mM of glucose (HG). (**e**) Scatter dot plots and arithmetic means ± SEM (*n* = 6; a.u.) of the NF-κB-dependent transcriptional activity in HAoSMCs following transfection with negative control siRNA (Negsi) or SGK1 siRNA (SGK1si) and treatment with control or 50 mM of glucose (HG). (**f**) Scatter dot plots and arithmetic means ± SEM (*n* = 6; a.u.) of the *ZFP36* relative mRNA expression in HAoSMCs following transfection with negative control siRNA (Negsi) or SGK1 siRNA (SGK1si) and treatment with control or 50 mM of glucose (HG). * *p* < 0.05, *** *p* < 0.001 significant vs. Negsi-transfected HAoSMCs; † *p* < 0.05, †† *p* < 0.01, ††† *p* < 0.001 significant vs. Negsi-transfected and HG-treated HAoSMCs.

The involvement of SGK1 in the high glucose-induced osteogenic transdifferentiation of HAoSMCs was confirmed by suppressing the endogenous expression of SGK1 using small interfering RNA (siRNA). As shown in Figure 4a, transfection with SGK1 siRNA significantly reduced the *SGK1* mRNA expression in HAoSMCs as compared to the negative control siRNA-transfected HAoSMCs. High glucose treatment significantly up-regulated the *SGK1* mRNA levels in negative control siRNA-transfected HAoSMCs (Figure 4a). High glucose-induced osteogenic marker mRNA expression and ALP activity were all significantly suppressed by SGK1 knockdown in HAoSMCs (Figure 4b–d). Moreover, the silencing of SGK1 blunted the high glucose-induced NF-κB activation and *ZFP36* mRNA expression and, thus, the NF-κB-dependent signaling in HAoSMCs (Figure 4e,f).

**Figure 5:**
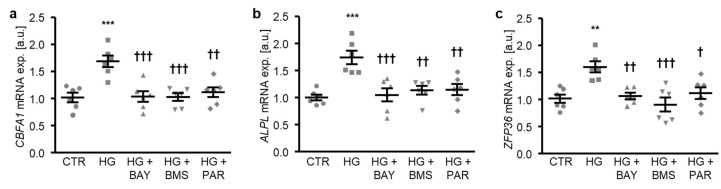
NF-κB inhibition reduces high glucose-induced osteogenic signaling in HAoSMCs. (**a**–**c**) Scatter dot plots and arithmetic means ± SEM (*n* = 6; arbitrary units, a.u.) of the *CBFA1* (**a**), *ALPL* (**b**), and *ZFP36* (**c**) relative mRNA expression in HAoSMCs following treatment with control (CTR) or 50 mM of glucose (HG) without or with 10 µM of BAY11-7082 (BAY), 10 µM of BMS-345541 (BMS), or 10 µM of parthenolide (PAR). ** *p* < 0.01, *** *p* < 0.001 significant vs. control HAoSMCs; † *p* < 0.05, †† *p* < 0.01, ††† *p* < 0.001 significant vs. HG-treated HAoSMCs.

To determine the critical role of NF-κB-dependent pathways in high glucose-induced osteogenic transdifferentiation, HAoSMCs were exposed to high glucose concentrations in the presence or absence of NF-κB pathway inhibitors: BAY11-7082, BMS-345541, or parthenolide. As illustrated in Figure 5a–c, each of the NF-κB inhibitors significantly suppressed high glucose-induced *CBFA1* and *ALPL*, as well as the *ZFP36* mRNA expression in HAoSMCs. Thus, NF-κB played a decisive role in the high glucose-induced osteogenic transdifferentiation of HAoSMCs.

**Figure 6:**
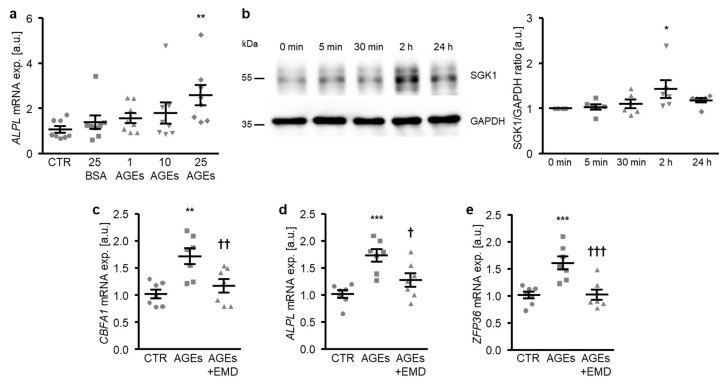
SGK1 inhibition suppresses AGEs-induced osteogenic signaling in HAoSMCs. (**a**) Scatter dot plots and arithmetic means ± SEM (*n* = 8; arbitrary units, a.u.) of the *ALPL* relative mRNA expression in HAoSMCs following treatment with control (CTR), 25 µg/mL of control BSA (BSA), or the indicated concentrations of AGE-BSA (AGEs; 1–25 µg/mL). (**b**) Representative original Western blots and scatter dot plots and arithmetic means ± SEM (*n* = 6; a.u.) of the normalized SGK1/GAPDH protein ratio in HAoSMCs following treatment for the indicated time (0–24 h) with 25 µg/mL of AGE-BSA. (**c**–**e**) Scatter dot plots and arithmetic means ± SEM (*n* = 7; a.u.) of the *CBFA1* (**c**), *ALPL* (**d**), and *ZFP36* (**e**) relative mRNA expression in HAoSMCs following treatment with control (CTR) or 25 µg/mL of AGE-BSA (AGEs) without or with 50 µM of SGK1 inhibitor EMD638683 (EMD). * *p* < 0.05, ** *p* < 0.01, *** *p* < 0.001 significant vs. control HAoSMCs; † *p* < 0.05, †† *p* < 0.01, ††† *p* < 0.001 significant vs. AGEs-treated HAoSMCs.

In the next experiments, the participation of SGK1-dependent signaling in the osteogenic transdifferentiation of HAoSMCs promoted by AGEs was investigated. To this end, HAoSMCs were exposed to AGEs of bovine serum albumin (BSA). As a result, the AGEs up-regulated the *ALPL* mRNA expression dose-dependently, the effects reaching statistical significance at 25 µg/mL AGEs concentration (Figure 6a). In contrast, 25 µg/mL of non-glycated BSA as a control did not significantly modify the *ALPL* mRNA expression as compared to control-treated HAoSMCs. Furthermore, the SGK1 protein abundance was significantly increased in HAoSMCs following 2 h of AGEs treatment (Figure 6b). Additional treatment with the SGK1 inhibitor EMD638683 significantly suppressed the AGEs-induced mRNA expression of osteogenic markers *CBFA1* and *ALPL* (Figure 6c,d) and of the NF-κB pathway downstream product *ZFP36* (Figure 6e). Thus, SGK1 participated in the osteoinductive signaling triggered by AGEs in HAoSMCs.

## 3. Discussion

The present study identified SGK1 as a critical regulator of the osteogenic transdifferentiation and calcification of VSMCs during hyperglycemic conditions. Previous studies already indicate an important role of SGK1 during phosphate-induced vascular calcification [30]. The current observations expand on the pro-calcific role of SGK1 during high glucose conditions, which could link vascular calcification in the distinct conditions of chronic kidney disease and diabetes mellitus. The SGK1 expression is up-regulated by high glucose and AGEs in VSMCs. The inhibition or knockdown of SGK1 interfere with osteoinductive signaling and, thus, reduce the osteogenic transdifferentiation and calcification of VSMCs promoted by high glucose. Similarly, SGK1 inhibition suppresses AGEs-induced osteogenic signaling in VSMCs.

In accordance with previous reports [16,17,39], the present observations show that high glucose directly promotes the osteogenic transdifferentiation of VSMCs, although relatively high glucose concentrations were required. In in vitro models of high glucose-induced VSMC calcification, the glucose levels required to induce a response in VSMCs presumably vary with the different origins of the cells and/or the cell culture medium used in experiments [16,17,39,40]. The effects of high glucose conditions were not mimicked by mannitol, which has been used as an osmotic control for high glucose-induced VSMC calcification processes [41,42]. This indicates a more potent activation of pro-calcific signaling pathways in VSMCs by glucose than hyperosmotic conditions, which requires further study to dissect the distinct signaling pathways during these conditions. However, it must be kept in mind that mannitol is not an ideal control, as it may have other effects such as hydroxyl scavenging [43] that may interfere with the osteoinductive pathways in VSMCs. In the model used in the present study, high glucose up-regulates the osteogenic marker expression dose-dependently, with a significant effect at 50 mM glucose supplementation. The high glucose-induced osteogenic transdifferentiation of VSMCs involves the increased expression of CBFA1, which further induces the production of osteogenic-specific proteins such as ALPL [24,44], key events in the initiation of vascular calcification [7,25]. By triggering the osteogenic transdifferentiation of VSMCs, high glucose promotes a pro-calcific environment in the vascular tissue [7]. Along these lines, high glucose enhances VSMC mineralization in vitro strongly in the presence of calcification medium. In vitro vascular calcification models require calcium and phosphate supplementation in the medium as substrates for calcification to permit maximal mineralization [45]. Accordingly, high glucose alone induces osteogenic transdifferentiation, but does not strongly affect the mineralization of VSMCs, as the substrate for mineralization may be insufficient. In addition, hyperglycemia impacts the osteogenic transdifferentiation of VSMCs [19] by inducing the formation of AGEs [18].

SGK1 mediates, at least partly, the osteoinductive effects of hyperglycemia in VSMCs. SGK1 triggers the osteo-/chondrogenic transdifferentiation of VSMCs [30], and SGK1 blockade is able to reduce vascular calcification [30,31,32]. We show here that SGK1 expression is increased in VSMCs by hyperglycemic conditions, while the inhibition or knockdown of SGK1 interferes with high glucose and AGEs-induced osteogenic transdifferentiation. Accordingly, the enhancement of VSMC mineralization induced by hyperglycemic conditions is blunted by SGK1 inhibition. Thus, SGK1 is required for the development of vascular calcification during hyperglycemia. However, the current study is limited to artificial cell culture conditions with high glucose levels, which may not be directly translatable to the situation in the human patient.

SGK1-downstream osteoinductive signaling involving NF-κB activation [30] participates in the vascular pro-calcific effects of hyperglycemia. In accordance with previous studies [35,36,37], NF-κB is activated by hyperglycemic conditions, while NF-κB interference suppresses the high glucose-induced osteogenic transdifferentiation of VSMCs. Furthermore, we show here that the NF-κB activation promoted by high glucose is SGK1-dependent. In addition to the key role in promoting vascular calcification [34], NF-κB activation may also augment apoptotic and inflammatory processes in the vascular tissue [35,46]. NF-κB plays, thus, an important role in vascular dysfunction [35]. Accordingly, NF-κB was suggested as a potential therapeutic target for vascular complications in diabetes [35]. However, SGK1 regulates many other cellular processes and signaling pathways and, thus, additional mechanisms may contribute to the pro-calcific role of SGK1 in VSMCs during hyperglycemic conditions. Further studies are required to elucidate the potential involvement of other mechanisms in the osteoinductive effects of SGK1 during these pathological conditions.

Taken together, SGK1 may play a crucial role in the development of vascular calcification in diabetic patients. In addition, SGK1 was shown to mediate the signaling promoting the osteo-/chondrogenic transdifferentiation of VSMCs and vascular calcification during other pathological conditions, such as disturbances in mineral homeostasis [30,31] or inflammation [32]. Thus, SGK1 may be a common regulator of vascular calcification promoted during various diseases and, thus, SGK1 inhibition may be a therapeutic option to reduce the progression of vascular calcification in large patient cohorts with distinct pathologies. Moreover, SGK1 inhibition may have additional protective effects during cardiovascular disease progression [47,48,49,50,51,52]. SGK1 may also play a role in the development of other complications, such as diabetic nephropathy [53,54,55,56]. Furthermore, SGK1 interference may impact glucose absorption and hyperglycemia [57]. Thus, SGK1 inhibition may have overall beneficial effects during disease progression in diabetes. Several SGK1 inhibitors were described: EMD638683 has been well characterized as an SGK1 inhibitor and has been used successfully in mice [58]. In addition, other SGK1 inhibitors have been developed and are being investigated [59,60].

## 4. Conclusions

During hyperglycemic conditions, SGK1 expression is up-regulated in VSMCs and SGK1-dependent signaling involving NF-κB pathway activation plays a key role in the osteogenic transdifferentiation and calcification of VSMCs. Thus, SGK1 inhibition may represent a treatment option to reduce the progression of vascular calcification promoted by hyperglycemia in diabetes mellitus.

## 5. Materials and Methods

### 5.1. Cell Culture

Primary human aortic smooth muscle cells (HAoSMCs; Fisher Scientific, Vienna, Austria and Sigma Aldrich, Vienna, Austria) were cultured in medium containing 1:1 ratio of Waymouth’s MB 752/1 and Ham’s F-12 nutrient mixture (~19 mM glucose, according to manufacturer’s information), 10% FBS, 100 U/mL of penicillin, and 100 µg/mL of streptomycin (all from Fisher Scientific, Vienna, Austria) [30,32,61,62,63] and used in experiments from passages 4 to 11.

HAoSMCs were treated with the indicated concentrations of glucose (Sigma Aldrich, Vienna, Austria), 50 mM of mannitol (Sigma Aldrich, Vienna, Austria), the indicated concentrations of AGE-BSA (stock in PBS, MBL International, Woburn, MA, USA), 25 µg/mL of control BSA (stock in PBS, MBL International, Woburn, MA, USA), 50 µM of SGK1 inhibitor EMD638683 (stock in DMSO, Biorbyt, Cambridge, UK) [30,32,49], 10 µM of BAY11-7082 (stock in DMSO, Sigma Aldrich, Vienna, Austria) [30], 10 µM of BMS-345541 (stock in DMSO, Sigma Aldrich, Vienna, Austria) [30], and 10 µM of parthenolide (stock in DMSO, Sigma Aldrich, Vienna, Austria) [30]. Equal amounts of vehicle were used as the control. HAoSMCs were treated with calcification medium containing 10 mM of β-glycerophosphate and 1.5 mM of CaCl_2_ (Sigma-Aldrich, Vienna, Austria) [33,61,62]. For long-term treatments, fresh medium with agents were added every 2–3 days.

HAoSMCs were transfected with negative control siRNA (ID no. 4390843, Fisher Scientific, Vienna, Austria) or 10 nM of SGK1 siRNA (ID no. s740, Fisher Scientific, Vienna, Austria) by using the siPORT amine transfection agent (Fisher Scientific, Vienna, Austria) [30,32] according to the manufacturer’s instructions. The silencing efficiency was analyzed by quantitative RT-PCR.

### 5.2. Quantitative RT-PCR

Total RNA was isolated from HAoSMCs after transfection and/or 24 h of treatment using Trizol Reagent (Fisher Scientific, Vienna, Austria) according to the manufacturer’s instructions. cDNA was synthesized with oligo(dT)_12–18_ primers (Fisher Scientific, Vienna, Austria) and SuperScript III Reverse Transcriptase (Fisher Scientific, Vienna, Austria). Quantitative RT-PCR was performed in duplicate with iQ^TM^ Sybr Green Supermix (Bio-Rad Laboratories, Vienna, Austria) and CFX96 Real-Time PCR Detection System (Bio-Rad Laboratories, Vienna, Austria). The following human primers were used (Fisher Scientific, Vienna, Austria, 5′→3′) [30,63,64]:

*ALPL* fw: GGGACTGGTACTCAGACAACG;
*ALPL* rev: GTAGGCGATGTCCTTACAGCC;
*CBFA1* fw: GCCTTCCACTCTCAGTAAGAAGA;
*CBFA1* rev: GCCTGGGGTCTGAAAAAGGG;
*GAPDH* fw: GAGTCAACGGATTTGGTCGT;
*GAPDH* rev: GACAAGCTTCCCGTTCTCAG;
*SGK1* fw: GCAGAAGAAGTGTTCTATGCAGT;
*SGK1* rev: CCGCTCCGACATAATATGCTT;
*ZFP36* fw: GACTGAGCTATGTCGGACCTT;
*ZFP36* rev: GAGTTCCGTCTTGTATTTGGGG.

The specificity of the PCR products was confirmed by the analysis of the melting curves. The relative mRNA expression was calculated by the 2^−ΔΔCt^ method using GAPDH as a housekeeping gene, normalized to the control group.

### 5.3. Protein Isolation and Western Blotting

After the indicated times (for time course experiments), 24 h (for total proteins) or 3 h (for phosphorylated proteins) of treatment, HAoSMCs were lysed with ice-cold IP lysis buffer (Fisher Scientific, Vienna, Austria) containing a complete protease and phosphatase inhibitor cocktail (Fisher Scientific, Vienna, Austria) [30,61,62], and the protein concentrations were determined by the Bradford assay (Bio-Rad Laboratories, Vienna, Austria). Equal amounts of proteins were boiled in Roti-Load1 Buffer (Carl Roth, Karlsruhe, Germany) at 100 °C for 10 min, separated on SDS-polyacrylamide gels, and transferred to PVDF membranes. The membranes were incubated with primary rabbit anti-RUNX2 (1:1000, #8486, Cell Signaling, Frankfurt am Main, Germany), rabbit anti-SGK1 (1:1000, #12103; Cell Signaling, Frankfurt am Main, Germany), rabbit anti-phospho-NDRG1 (Thr^346^) (1:1000, #3217; Cell Signaling, Frankfurt am Main, Germany), or rabbit anti-GAPDH (1:1000, #2118, Cell Signaling, Frankfurt am Main, Germany) antibodies overnight at 4 °C and then with secondary anti-rabbit HRP-conjugated antibody (1:1000, Cell Signaling, Frankfurt am Main, Germany) for 1 h at room temperature. The membranes were stripped in stripping buffer (Fisher Scientific, Vienna, Austria) for 10 min at room temperature. Antibody binding was detected with ECL detection reagent (Fisher Scientific, Vienna, Austria). Bands were quantified using the ImageJ software and the results are shown as the ratio of total protein to GAPDH or phosphorylated protein to GAPDH, normalized to the control group.

### 5.4. Quantification of Calcification

For the quantification of calcification following treatment for 11 days, HAoSMCs were decalcified in 0.6 M of HCl overnight at 4 °C and the calcium content was determined using the QuantiChrom Calcium assay kit (BioAssay Systems, Hayward, CA, USA) according to the manufacturer’s instructions [32,65,66]. HAoSMCs were lysed with 0.1 M of NaOH/0.1% SDS and the protein concentrations were measured by the Bradford assay (Bio-Rad Laboratories, Vienna, Austria). The results are shown normalized to the total protein concentration.

### 5.5. ALP Activity Assay

HAoSMCs were lysed with ALP Assay buffer after transfection and/or 7 days of treatment and the ALP activity was determined by using the ALP colorimetric assay kit (Abcam, Cambridge, UK) [63,67] according to the manufacturer’s instructions. Protein concentrations were measured by the Bradford assay (Bio-Rad Laboratories, Vienna, Austria) and the results are shown normalized to the total protein quantity and to the control group.

### 5.6. NF-κB Activity Assay

Nuclear extracts were isolated from HAoSMCs following transfection and/or 30 min of treatment using the NE-PER nuclear and cytoplasmic extraction reagents (Fisher Scientific, Vienna, Austria) [30,65] according to the manufacturer’s instructions, and the protein concentrations were determined by the Bradford assay (Bio-Rad Laboratories, Vienna, Austria). Equal amounts of nuclear proteins were used to determine the NF-κB transcriptional activity with the NF-κB p65 transcription factor colorimetric assay kit (Abcam, Cambridge, UK) according to the manufacturer’s instructions. Results are shown normalized to the control group.

### 5.7. Statistics

Data are shown as scatter dot plots and arithmetic means ± SEM and *n* indicates the number of independent experiments performed at different cell passages. Statistical analysis was performed using the SPSS and JMP software. Normality was tested with a Shapiro–Wilk test. Non-normal datasets were transformed (log, sqrt, or reciprocal) prior to statistical testing to provide normality according to the Shapiro–Wilk test. Statistical testing was performed by a one-way ANOVA followed by Tukey’s HSD test (homoscedastic data) or the Games–Howell test (heteroscedastic data). Non-normal data were tested by the Steel–Dwass method. Two groups were compared by the unpaired two-tailed t-test. *p* < 0.05 was considered statistically significant.
